# Exogenous Allantoin Confers Rapeseed (*Brassica campestris*) Tolerance to Simulated Drought by Improving Antioxidant Metabolism and Physiology

**DOI:** 10.3390/antiox12081508

**Published:** 2023-07-27

**Authors:** Md. Rakib Hossain Raihan, Mira Rahman, Anshu Rastogi, Masayuki Fujita, Mirza Hasanuzzaman

**Affiliations:** 1 Department of Agronomy, Faculty of Agriculture, Sher-e-Bangla Agricultural University, Sher-e-Bangla Nagar, Dhaka 1207, Bangladesh; 2 Laboratory of Bioclimatology, Department of Ecology and Environmental Protection, Poznań University of Life Sciences, Piątkowska 94, 60-649 Poznan, Poland; 3 Faculty of Agriculture, Kagawa University, Kita-Gun, Kagawa, Miki-cho 761-0795, Japan; 4 Kyung Hee University, 26 Kyungheedae-ro, Dongdaemun-gu, Seoul 02447, Republic of Korea

**Keywords:** antioxidant defense system, ascorbate–glutathione pathway, hydrogen peroxide, stress elicitor, water stress, ureides

## Abstract

Allantoin is an emerging plant metabolite, but its role in conferring drought-induced oxidative stress is still elusive. Therefore, an experiment was devised to explore the role of allantoin (0.5 and 1.0 mM; foliar spray) in rapeseed (*Brassica campestris* cv. BARI Sarisha-17) under drought. Seedlings at fifteen days of age were subjected to drought, maintaining soil moisture levels at 50% and 25% field capacities, while well-irrigated plants served as the control group. Drought-stressed plants exhibited increased levels of lipid peroxidation and hydrogen peroxide, electrolyte leakage, and impaired glyoxalase systems. Thus, the growth, biomass, and yield attributes of rapeseed were significantly impaired under drought. However, the allantoin-supplemented plants showed a notable increase in their contents of ascorbate and glutathione and decreased dehydroascorbate and glutathione disulfide contents under drought. Moreover, the activity of antioxidant enzymes such as ascorbate peroxidase, dehydroascorbate reductase, glutathione reductase, glutathione peroxidase, and catalase were accelerated with the allantoin spray and the glyoxalase system was also enhanced under drought. Moreover, the improvement in water balance with reduction in proline and potassium ion contents was also observed when allantoin was applied to the plants. Overall, the beneficial effects of allantoin supplementation resulted in the improved plant growth, biomass, and yield of rapeseed under drought conditions. These findings suggest that allantoin acts as an efficient metabolite in mitigating the oxidative stress caused by reactive oxygen species by enhancing antioxidant defense mechanisms and the glyoxalase system.

## 1. Introduction

Plants are frequently exposed to various adverse environments, such as drought, waterlogging, salinity, extreme temperatures, and others, due to rapid climate changes. These environmental conditions exert a disruptive effect on plant physiological and biochemical processes, which in turn reduce plant growth and productivity [1]. Among these stressors, drought is considered the most significant threat to agriculture and crop production. Irregular atmospheric circulation, rising temperatures, rapid surface evaporation, and loss of natural precipitation are major factors contributing to the widespread occurrence of drought worldwide [2]. Around 64% of lands across the globe have been estimated as drought-prone areas and are accountable for reducing crop yield by up to 50% [1,3]. Drought creates obstacles to obtaining water and nutrients for plants and leads to cellular dysfunction [4]. It also accelerates leaf senescence, impairs photosynthetic apparatuses, reduces carbon dioxide fixation, and affects assimilate translocation rate in plants [5,6]. Moreover, dehydration in tissues induces metabolic injury [7], and reduction in leaf relative water content (RWC) and stomatal conductance in plants ultimately impair growth and biomass production [8]. Another abrupt consequence of drought is imbalance in the antioxidant defense system and reactive oxygen species (ROS) metabolism. Plants’ antioxidant defense system is composed of enzymatic and non-enzymatic antioxidants that are combinedly employed in the detoxification of over-generated ROS under stress. But when an imbalance occurs in the antioxidant defense system in plants, it leads to the overaccumulation of ROS, i.e., hydrogen peroxide (H_2_O_2_), singlet oxygen (^1^O_2_); hydroxyl radical (^•^OH), superoxide anion radical (O_2_^•−^), etc. [9]. The plasma membrane, apoplast, cytosols, chloroplasts, peroxisomes, mitochondria, and endoplasmic reticulum are the major sites of ROS generation [10]. These ROS molecules are highly reactive and can bind to various biomolecules, including carbohydrates, lipids, proteins, nucleic acids, vitamins, and hormones, inducing oxidative stress by oxidizing lipids, denaturing proteins, and mutating nucleic acids [11,12]. Moreover, drought also accelerates the accumulation of methylglyoxal (MG), which is a reactive α-ketoaldehyde formed during glucose metabolism [13]. This glycating compound reacts with proteins, lipids, and nucleic acids, leading to the formation of glycation end products that hinder normal metabolic activities and damage cellular organelles [14]. A plant’s inherent mechanisms involving antioxidant defense against ROS and MG detoxification, osmoprotectant accumulation, etc., help elucidate the damage caused by drought [14,15].

Allantoin is a heterocyclic nitrogenous product that was first extracted from *Symphytum officinale* by Macalister [16]. The structural formula of allantoin is 1-(2,5-dioxoimidazolidin-4-yl) urea, popularly known as 5-ureidohydantoin. Allantoin and its hydrolyzed form (allantoate) are popularly called ureides, and these are the key forms of nitrogenous molecules in plants that are carried from source to sink [17]. Ureides are more efficient in nitrogen transport than amide forms (glutamine and asparagine) due to their lower carbon-to-nitrogen (C:N) ratio and higher potentiality to release ammonium ions upon the breakdown of allantoin. Ureides constitute around 90% of the total nitrogenous compounds in legumes, whereas they only account for 15% of the total nitrogenous compounds in non-leguminous plants [18]. Allantoin performs an intermediate role in the pathway of purine catabolism by storing and reusing nitrogen in plants [19]. Previous reports showed that allantoin application increased the yield of crops [20]. Recent studies on allantoin reported that its accumulation increases in plant cells under various stresses such as drought [21,22], salinity [23], metal toxicity [24], high irradiance [25], and low temperature [26]. An increase in allantoin levels can modulate different stress responses in plants. In rice, the expression of ureide permease1 was enhanced under nitrogen-deficient conditions, leading to increased allantoin accumulation in plants. This, in turn, has been reported to improve the growth of rice plants, as observed by Redillas et al. [27]. Allantoin can act on the abscisic acid signaling pathway and improve ROS metabolism to increase salt tolerance in *Arabidopsis thaliana* [28]. Dresler et al. [24] suggested that allantoin increased ascorbate (AsA) and glutathione (GSH) levels and indirectly employed ROS scavenging in cucumber plants under cadmium stress. This evidence is good enough to mention that allantoin accumulation during stress can be an effective modulator of stress in plants.

Rapeseed (*Brassica campestris*) is one of the most economically valuable oilseed crops in the world, belonging to the Brassicaceae family. It is nutritionally most viable for its profound amounts of amino acids, essential vitamins, and minerals and also for having healthy fatty acids [29]. Rapeseed is highly susceptible to drought, and the occurrence of drought at any stage of the life span could detrimentally impact the growth and oil content of these plants [30]. Water-deficit conditions adversely affect rapeseed’s physiological processes, viz., photosynthetic rates, osmotic protection, etc., and stunt plant growth [31]. The degree of drought stress tolerance depends on the rapeseed’s growth stage and crop species. Plants evolve an array of morphological, physiochemical, and molecular mechanisms to adapt and survive under stress conditions [32]. The exogenous application of osmolytes such as trehalose [33] and glycine betaine [34]; plant growth regulators such as jasmonic acid [35] and salicylic acid [36]; and plant nutrients such as potassium [37], calcium [38], etc., show better tolerance to drought. However, there are hardly any studies regarding the role of allantoin in conferring plant drought tolerance; in particular, studies emphasizing ROS metabolism have not been reported yet. Moreover, allantoin is involved in purine metabolism with regard to storing and using nitrogen in plants [19]. Therefore, the present study aimed to investigate allantoin’s role in ROS scavenging and the antioxidant defense system under drought. Particularly, we intended to check whether allantoin (i) can reduce ROS-induced damage in plants caused by drought, (ii) can modulate the antioxidant activities of plants under stress, (iii) can regulate the osmotic imbalances of plants, and (iv) can improve the growth and yield attributes of rapeseed under drought. Furthermore, the successful accomplishment of our study provides insight into the mechanisms of allantoin in the drought tolerance of rapeseed.

## 2. Materials and Methods

### 2.1. Plant Materials and Stress Exposure

**Figure 1:**
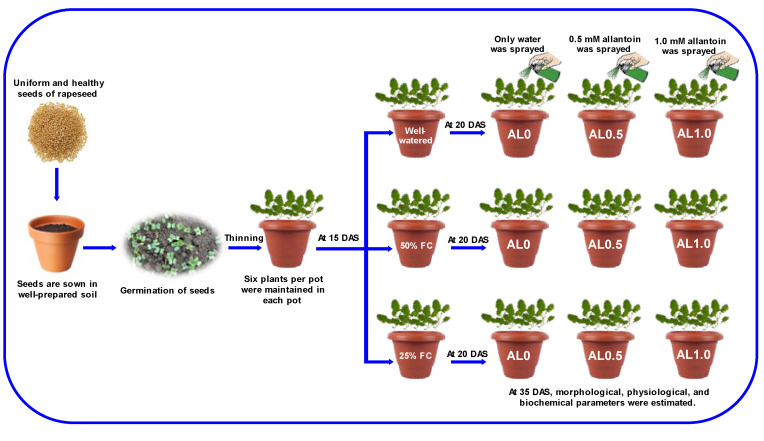
Schematic representation of experimental procedure and treatment details. Here, FC: field capacity, AL: allantoin. DAS: Days after sowing.

Seeds of *Brassica campestris* cv. BARI Sarisha-17 were used as the plant material to execute this study. Matured and uniform-sized seeds were sterilized first, then sown in plastic pots (14 L). The seeds of BARI Sarisha-17 were collected from Bangladesh Agricultural Research Institute (BARI), Gazipur, Bangladesh. This experiment was set in a shed house where the average day and night temperature were about 26 ± 5 °C and 17 ± 4 °C, respectively. The relative humidity varied around 53 ± 5%, and the photosynthetic photon flux density (PPFD) was about 800–2300 μmol m^–2^ s^–1^. During final soil preparations, BARI [39]-recommended fertilizer doses were incorporated into the soils properly and each pot was filled with 13 kg of soil. The field capacities of two levels of drought, i.e., 50% and 25%, were maintained, and the soil moisture level was measured using a soil moisture meter (Model no: WH0291) from 15 days after sowing (DAS), whereas regular irrigation was performed to the control. The foliar spray of allantoin (C_4_H_6_N_4_O_3_; FujiFilm Wako Pure Chemical Corporation, Osaka, Japan) was initiated from 20 DAS at 0.5 and 1.0 mM concentrations, which were indicated as AL0.5 and AL1.0, respectively, while only distilled water (dH_2_O) was sprayed on the AL0-indicated plants at 3-day intervals (Figure 1). In the preliminary trial, it was observed that allantoin concentrations above 1.0 mM had some toxic effects on rapeseed, while concentrations below 0.5 mM did not yield remarkable responses. Although we did not measure the specific concentration of allantoin in the seedlings, previous studies by other authors have indicated an increase in its endogenous concentration in other plants after foliar spray [28,40]. Based on this information, we assumed that applying allantoin at 0.5 and 1.0 mM concentrations would provide sufficient availability for the rapeseed plants. To execute the study, a completely randomized design (CRD) was followed where two sets of pots were maintained, one for growth and yield attributes and another one for physiological and biochemical attributes. Different parameters of morphophysiological and biochemical parameters of rapeseed plants were taken at 35 DAS and harvesting was performed at 90 DAS for measuring the yield attributes.

### 2.2. Measurement of Growth Attributes

Five plants from each treatment were selected randomly for estimating plant height. Using a scale, the height of the plants was measured and averaged. The SPAD reading was taken from five fully expanded leaves using a SPAD meter (FT Green LLC, Wilmington, DE, USA). The obtained values were then averaged to estimate the SPAD value for chlorophyll (Chl) content. Five plants from each treatment were uprooted for estimating fresh weight (FW) and dry weight (DW). Immediately after uprooting, the weight of the plants was taken with an electric balance and averaged to estimate the FW. After that, sun drying of the plant samples was performed to reduce the initial moisture, followed by oven-drying at 80 °C for 72 h. Then, the weight of the plants was taken and averaged for the DW. For partitioning FW and DW, three plants were uprooted randomly from each treatment. Then, the stem, leaf, and siliqua were separated and weighted individually for FW. Hereafter, the samples were sun-dried for a couple of days, followed by oven-drying at 80 °C for 72 h. Then, the weight of the dried samples was taken individually for the DW and averaged.

### 2.3. Estimation of Relative Water Content

The method ascribed to Barrs and Weatherly [41] was followed to estimate the RWC, where fully expanded and same-aged three-leaf laminas were excised from each treatment, immediately after which the FW was taken. Then, the laminas were immersed in dH_2_O for 24 h, covered with filter paper (two layers), and kept in the dark. After 24 h, the excess moisture was removed with blotter paper with a slight press and weighed for the turgid weight (TW). Finally, these laminas were over-dried for 48 h at 80 °C and the DW was taken. Using the fresh, turgid, and dry weight values, the following equation was followed to estimate the RWC:

$$RWC\;(\%)=\frac{FW\;-\;DW}{TW\;-\;DW}\times 100$$

### 2.4. Determination of Proline Content

Following the procedure outlined by Bates et al. [42], fresh leaves weighing 0.5 g were macerated with 5 mL of 3% aqueous sulfosalicylic acid in a chilled mortar and pestle. The mixture was then centrifuged at 12,000× *g* for 12 min. In a 15 mL Falcon tube, a combination of the extracted supernatant, acid ninhydrin in 6 M phosphoric acid, and glacial acetic acid was mixed in a ratio of 1:1:1. This mixture was incubated for 60 min in a water bath set at 100 °C. Hereafter, the incubated mixture was thoroughly cooled in an ice bath and 4 mL of toluene was added to it. Toluene separated free proline from the mixture. The spectrophotometric reading at 520 nm was then taken for the separated chromophore. To determine the actual concentration of proline in the leaves, a standard curve using known proline concentrations was prepared.

### 2.5. Estimation of K^+^ Content

The K^+^ ion content was measured using a compact K^+^ meter (Laquatwin-Ca-11, HORIBA Advanced Techno Co., Romeoville, IL, USA). The cell sap of the harvested leaf was extracted using a sap extractor, and drops of cell sap were applied to the sensor of the ion meter. After a certain period of time, a smiley sign appeared on the screen, indicating the value that corresponded to the concentration of ions present in the sample.

### 2.6. Determination of Electrolyte Leakage

To estimate leaf EL, in a Falcon tube, fresh and chopped leaf (0.5 g) and dH_2_O (15 mL) were taken following the procedure of Dionisio-Sese and Tobita [43]. This mixture was then heated for 40 min in a water bath at 60 °C and the first electrical conductivity (EC_1_) of the solution was measured using a HI-993310 electrical conductivity meter (Hanna, Woonsocket, RI, USA). The following mixture was again heated for the second conductivity (EC_2_) at 121 °C in an autoclave for 20 min. Then the value of EC_1_ divided by EC_2_ and multiplied by 100 following the equation below was used to calculate the leaf EL. The formula is presented below:

$$EL\;(\%)=\frac{{EC}_{1}}{{EC}_{2}}\times 100$$

### 2.7. Quantification of Malondialdehyde Content

Heath and Packer [44] proposed a mechanism to estimate the degree of lipid peroxidation as malondialdehyde (MDA) content. Their procedure is based on the thiobarbituric acid (TBA) method for which half of a gram of fresh leaf was ground properly, adding 5% trichloroacetic acid (*w*/*v*; 3 mL). The homogenization was then centrifuged for 12 min at 12,000× *g* to extract a clear supernatant that was mixed with 4 mL of 0.5% TBA and 20% trichloroacetic acid solution. The reaction of the TBA reagent with the plant sample will be accomplished after heating for 30 min in a water bath. Rapid cooling of the boiled mixture was needed before taking the absorbances at 532 and 600 nm in a spectrophotometer. The non-specific absorbance (600 nm) reading was then subtracted from that at 532 nm and the MDA content was calculated using an extinction coefficient of 155 m M^–1^ cm^–1^.

### 2.8. Estimation of Hydrogen Peroxide Content

The proposed procedure of Yu et al. [45] was followed to determine the H_2_O_2_ content. A fresh leaf of 0.5 g was homogenized with 5% (*w*/*v*) trichloroacetic acid and centrifuged for 12 min at 12,000× *g*. Subsequently, the supernatant was combined with potassium iodide (1 mM), and potassium phosphate (K-P) buffer of pH 7.0 (10 mM) at the ratio of 1:2:1. The mixture was then incubated for 60 min. The density of the resulting mixture was measured spectrophotometrically at 390 nm, and the H_2_O_2_ content was calculated using a standard curve.

### 2.9. Estimation of Ascorbate and Glutathione Content

Half a gram of fresh leaf was finely ground with 3 mL of a reagent prepared with metaphosphoric acid and ethylenediaminetetraacetic acid (EDTA). The homogenate was followed by centrifugation (12,000× *g*, 15 min) for supernatant. For reduced AsA, 200 μL of supernatant was neutralized with K-P buffer (0.5 M, pH 7.0) and dH_2_O, and the activity was assayed with K-P buffer (100 mM, pH 6.5) and ascorbate oxidase (AO; 0.5 U) at 265 nm in a spectrophotometer. For total AsA, it was neutralized with dithiothreitol (DTT, 0.1 M) instead of H_2_O, and spectrophotometrically observed with K-P buffer and AO. The contents of reduced AsA and total AsA were determined by plotting the values against a standard curve, and the dehydroascorbate (DHA) content was estimated as follows: total AsA–reduced AsA [46].

For total GSH, the extract was neutralized using K-P buffer (0.5 M, pH 7.0) and dH_2_O. Then, the activity was observed at 412 nm in a spectrophotometer after oxidizing the neutralized sample with 5,5′-dithio-bis(2-nitrobenzoic acid) (DTNB) and reduced with nicotinamide adenine dinucleotide phosphate (NADPH) and glutathione reductase (GR). In the case of glutathione disulfide (GSSG), the extract was neutralized with 2–vinylpyridine instead of dH_2_O and spectrophotometrically observed. Then, the total GSH and GSSG content was measured by plotting against a standard curve and the GSH content was calculated as follows: total GSH–GSSG [45].

### 2.10. Enzyme Extraction, Protein Determination, and Antioxidant Enzyme Activities

Fresh leaf (0.5 g) was extracted with 1 mL of extraction buffer, prepared with L-ascorbic acid (Asc) (1 mM), K-P buffer (50 mM, pH 7.0), KCl (100 mM), β-mercaptoethanol (5 mM), and glycerol (10%), following the procedure of Hasanuzzaman et al. [47]. Then, the homogenate was centrifuged at 12,000× *g* for 15 min and the supernatant was preserved for protein determination and antioxidant enzyme activities.

For protein determination, the procedure of Bradford [48] was followed. Bradford reagent was prepared with Coomassie brilliant blue (CBB G-250), ethanol (100%), ortho-phosphoric acid (85%), and dH_2_O. Then, 5 mL Bradford reagent was mixed with 5 μL of the supernatant, then spectrophotometrically observed at 595 nm. Then, the protein concentration was estimated with a standard curve made with bovine serum albumin.

The activities of APX, DHAR, GR, GPX, CAT, Gly I, and Gly II were observed spectrophotometrically following the assay buffer, and using an extinction coefficient, their activity was calculated, depicted in Table 1.

**Table 1:** Preparations of assay buffer, spectrophotometer wavelength, and extinction coefficient for the determination of enzyme activity.

Names of the Enzymes	Name of the Chemicals and Their Concentrations	Wavelength	Extinction Coefficient	References
Ascorbate peroxidase (APX; EC: 1.11.1.1)	L-ascorbic acid (Asc; 0.5 mM) Ethylenediaminetetraacetic acid (EDTA; 0.1 mM) Potassium phosphate (K-P) buffer (15 mM, pH 7.0) Hydrogen peroxide (H_2_O_2_; 0.1 mM)	290 nm	2.8 mM^−1^ cm^−1^	[49]
Dehydroascobate reductase (DHAR; EC: 1.8.5.1)	Reduced glutathione (GSH; 2.5 mM) EDTA (0.1 mM) K-P buffer (50 mM, pH 7.0) Dehydroascorbate (DHA; 0.1 mM)	265 nm	14 mM^−1^ cm^−1^	[49]
Glutathione reductase (GR; EC: 1.6.4.2)	Oxidized glutathione (GSSG; 1 mM) Nicotinamide adenine dinucleotide phosphate (NADPH; 0.2 mM) EDTA (1 mM) K-P buffer (0.1 M, pH 7.8)	340 nm	6.2 mM^−1^ cm^−1^	[50]
Glutathione peroxidase (GPX; EC: 1.11.1.9)	NADPH (0.12 mM) GR (1 unit) GSH (2 mM) Sodium azide (1 mM) EDTA (1 mM) K-P buffer (100 mM, pH 7.0)	340 nm	6.62 mM^−1^ cm^−1^	[50,51]
Catalase (CAT; EC: 1.11.1.6)	H_2_O_2_ (15 mM) K-P buffer (50 mM, pH 7.0)	240 nm	39.4 M^−1^ cm^−1^	[50]
Glyoxalase I (Gly I; EC: 4.4.1.5)	GSH (100 mM) Magnesium phosphate (16 mM) Methylglyoxal (100 mM) Sodium phosphate buffer (0.1 M)	240 nm	3.37 mM^−1^ cm^−1^	[50]
Glyoxalase II (Gly II; EC: 3.1.2.6)	5,5′-dithio-bis(2-nitrobenzoic acid) (DTNB; 0.2 mM) *S*-_D_-lactoylglutathione (1 mM) Tris-HCl buffer (100 mM, pH 7.2)	412 nm	13.6 mM^−1^ cm^−1^	[52]

### 2.11. Yield and Yield Attributes

The number of siliques was counted manually from five plants and averaged. To estimate the silique length, ten siliques from each treatment were selected, and the length was measured with a scale and averaged. For the determination of the number of seeds silique^−1^, the seeds of ten randomly selected siliques were counted and averaged. With an electric seed counter, one thousand seeds were computed and weighed to determine the 1000-seed weight. The total weight of the seeds of five plants was weighed and averaged to determine the seed yield of plant^−1^.

### 2.12. Statistical Analysis

The mean (±SD) value was computed from three (n = 3) replications. Different parameters were compared among the treatments using Tukey’s HSD test at *p* ≤ 0.05. The statistical analysis was accomplished with the computer-based software CoStat v.6.400 and subjected to two-way analysis of variances to analyze the effect of different water regimes (WRs) and allantoin application (CoHort Software, Monterey, CA, USA) [53]. Correlation analysis was performed considering the 1% level of significance by using SPSS v.27 [54].

## 3. Results

### 3.1. Allantoin Enhances Relative Water Availability, Maintains Osmoregulation, and Regulates Potassium Ion Accumulation in Plants under Drought

**Figure 2:**
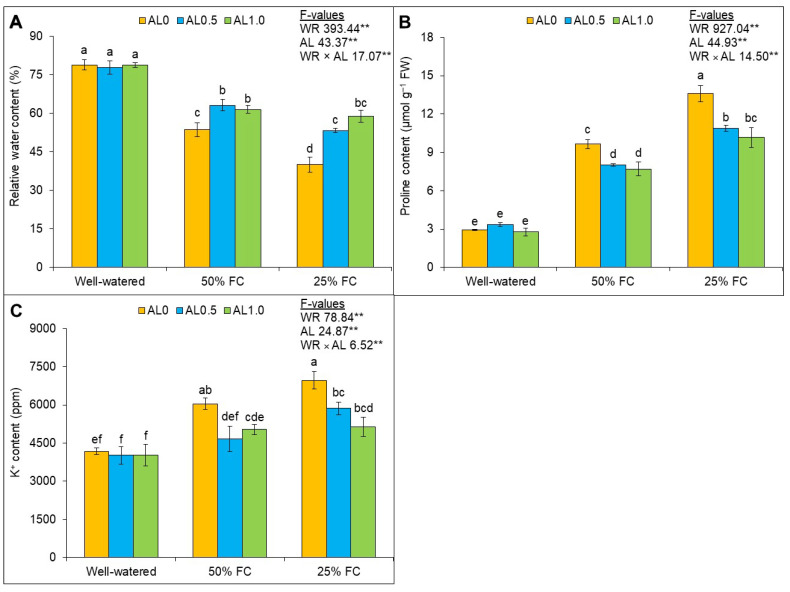
Changes in the (**A**) relative water content, (**B**) proline content, and (**C**) K^+^ content of rapeseed under drought. Here, AL0, AL0.5, and AL1.0 indicate 0, 0.5, and 1.0 mM allantoin, respectively. Mean (±SD) value calculated from three replications and different letters on each bar indicate significant differences among the treatments at *p* ≤ 0.05 after applying Tukey’s HSD test. **: *p* < 0.01, WR: water regime, AL: allantoin.

The RWC was reduced significantly (*p* < 0.01) by drought. The allantoin application significantly (*p* < 0.01) increased the RWC of the plants. The RWC of the plants was reduced by 25 and 39% under mild and severe drought conditions, respectively, compared to the control group. The application of 0.5 mM allantoin (10 and 8%) and 1.0 mM allantoin (13% and 19%) further increased the RWC of plants under mild and severe drought (Figure 2A). The effects of drought increased proline content significantly (*p* < 0.01). However, the application of allantoin to the drought-affected plants showed a significantly lower level of proline content at *p* < 0.01. The proline accumulation was shown to increase by 228% and 362% under mild and severe drought, respectively, compared to the control. Under mild drought, the proline content was decreased by 17%, and a 25% reduction in proline was found under severe drought (Figure 2B). The effect of drought, allantoin application, and their interaction were significant (*p* < 0.01). When plants were exposed to mild and severe drought, the K^+^ ion content was also increased by 45% and 67%, respectively, over control. The concomitant decreases in K^+^ content in response to 0.5 mM and 1.0 mM allantoin were found to be 23% and 17%, respectively, under mild drought, and 16% and 26%, respectively, under severe drought (Figure 2C).

### 3.2. Exogenous Allantoin Protects ROS-Induced Cellular Damage and Increases Membrane Stability in Plants under Drought

**Figure 3:**
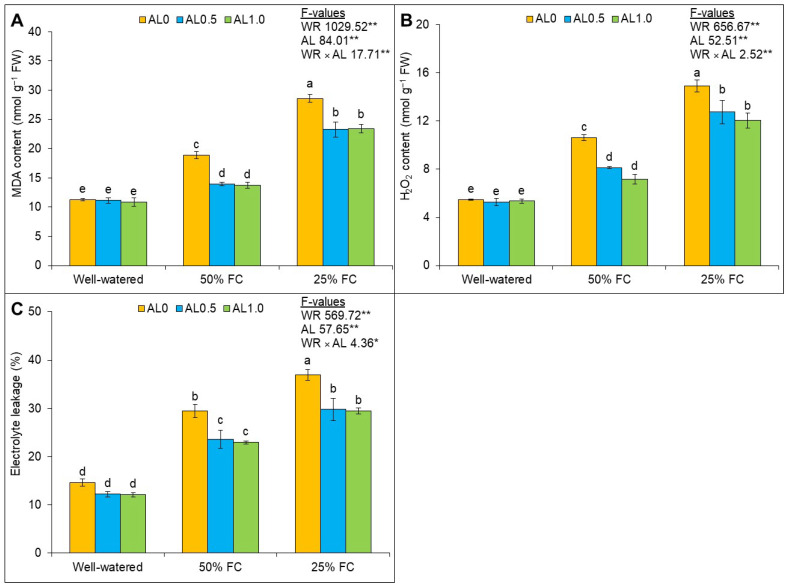
Changes in the contents of (**A**) malondialdehyde (MDA), (**B**) hydrogen peroxide (H_2_O_2_), and (**C**) electrolyte leakage of rapeseed under drought. Here, AL0, AL0.5, and AL1.0 indicate 0, 0.5, and 1.0 mM allantoin, respectively. Mean (±SD) value calculated from three replications and different letters on each bar indicate significant differences among the treatments at *p* ≤ 0.05 after applying Tukey’s HSD test. **: *p* < 0.01, *: *p* < 0.05, WR: water regime, AL: allantoin.

The effect of drought, allantoin, and their interactions were significant (*p* < 0.01) on the MDA, H_2_O_2_, and EL of the plants. Both mild and severe drought caused an increase in the content of MDA by 67% and 153%, respectively, over the control. Allantoin application, however, at 0.5 mM and 1.0 mM concentrations reduced the MDA content by 26% and 27% under mild drought; in contrast, MDA content was decreased by 19% and 18%, respectively, under severe drought compared to the mild drought condition (Figure 3A). A notable increase in H_2_O_2_ content under mild and severe drought was observed, 94% and 173%, respectively, compared to the control. However, the content of H_2_O_2_ was reduced with the application of 0.5 mM allantoin by 23% and 33% under mild drought, whereas it was decreased by 14% and 19% with 1.0 mM allantoin under severe drought (Figure 3B). A similar trend of the increase in the percentage of EL was also observed under mild and severe drought by 15% and 22%, respectively, compared to the control. However, the supplementation of allantoin notably reduced the EL of the plants under drought (Figure 3C).

### 3.3. Allantoin Regulates AsA-GSH Pool under Drought

**Figure 4:**
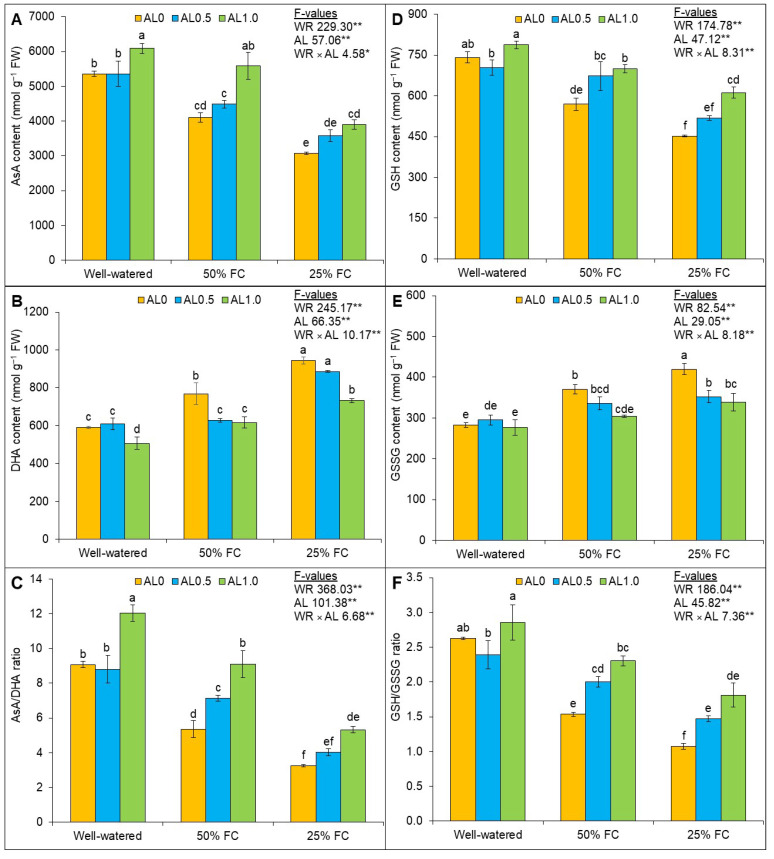
Changes in the (**A**) ascorbate (AsA) and (**B**) dehydroascorbate (DHA) contents, (**C**) AsA/DHA ratio, (**D**) glutathione (GSH) and (**E**) glutathione disulfide (GSSG) contents, and (**F**) GSH/GSSG ratio of rapeseed under drought. Here, AL0, AL0.5, and AL1.0 indicate 0, 0.5, and 1.0 mM allantoin, respectively. Mean (±SD) value calculated from three replications and different letters on each bar indicates significant differences among the treatments at *p* ≤ 0.05 after applying Tukey’s HSD test. **: *p* < 0.01, *: *p* < 0.05, WR: water regime, AL: allantoin.

The effects of drought and allantoin were significant (*p* < 0.01) on the AsA content. The interaction of factors also affected the AsA content significantly (*p* < 0.05). The changes in AsA and AsA/DHA ratio were significantly affected (*p* < 0.01) by drought and allantoin application. Mild and severe drought caused reductions in AsA content by 24% and 43%, respectively, compared to the control, whereas the DHA content was increased by 30% and 60%, respectively. Thus, the ratio of AsA/DHA was adversely affected, and it decreased by 41% and 64% under mild and severe drought, respectively, compared to the control. However, allantoin application at 1.0 mM notably increased the AsA content (36%) with a concomitant decrease in the DHA content; therefore, the ratio of AsA/DHA was also enhanced by 70% by the application of allantoin under mild drought in comparison to the only drought conditions with no application of allantoin. Similarly, under severe drought, the application of 1.0 mM allantoin uplifted the AsA content and AsA/DHA ratio and reduced DHA content (Figure 4A–C).

The effect of drought, allantoin application, and their interaction were found to be significant (*p* < 0.01) on the GSH, GSSG, and GSH/GSSG ratio of the plants. In the case of GSH content, it was decreased by 23% and 39% when subjected to mild and severe drought, respectively, whereas 31% and 49% increases, respectively, in the GSSG content were observed; thus, the ratio of GSH/GSSG ultimately reduced when subjected to mild and severe drought. Allantoin application at 1.0 mM to the mild- and severe-drought-affected plants notably uplifted the GSH content by 23% and 35%, respectively, and decreased the GSSG content by 18% and 19%, respectively; thus, the ratio of GSH/GSSG increased in the plants under drought conditions (Figure 4D–F).

### 3.4. Allantoin Causes Enhanced Antioxidant Activities and Boosts Drought Tolerance of Plants

**Figure 5:**
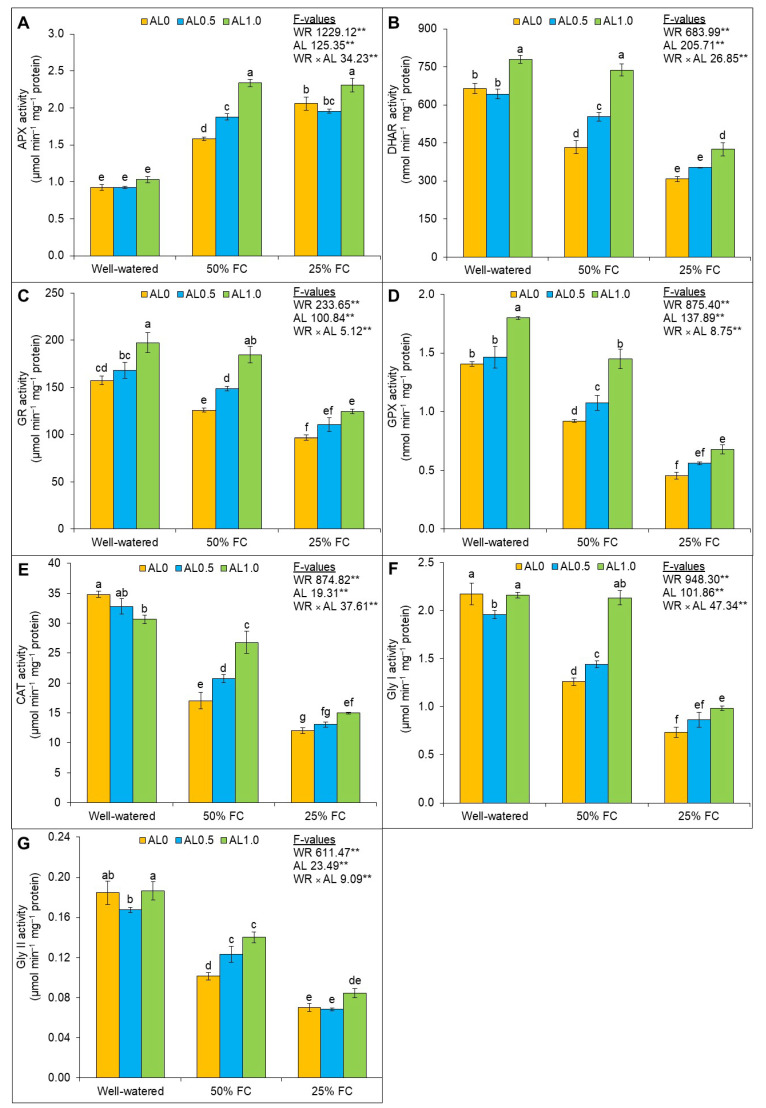
Changes in the activities of (**A**) ascorbate peroxidase (APX), (**B**) dehydroascorbate reductase (DHAR), (**C**) glutathione reductase (GR), (**D**) glutathione peroxidase (GPX), (**E**) catalase (CAT), (**F**) glyoxalase I (Gly I), and (**G**) glyoxalase II (Gly II) of rapeseed under drought. Here, AL0, AL0.5, and AL1.0 indicate 0, 0.5, and 1.0 mM allantoin, respectively. Mean (±SD) value calculated from three replications and different letters on each bar indicate significant differences among the treatments at *p* ≤ 0.05 after applying Tukey’s HSD test. **: *p* < 0.01 WR: water regime, AL: allantoin.

The changes in the activities of APX, DHAR, GR, GPX, and CAT were significant (*p* < 0.01) and caused by either drought or allantoin application. The APX activity was increased, whereas the activities of DHAR, GR, GPX, and CAT were reduced upon exposure to mild and severe drought compared to the control. However, the highest increase in APX activity was found with 1.0 mM allantoin, and it was by 48% and 12% under mild and severe drought, respectively, in comparison to the corresponding drought only (Figure 5A). Supplementation with 0.5 mM and 1.0 mM allantoin enhanced the DHAR activity by 28% and 70%, respectively, under mild drought compared to the effect of drought alone, whereas it only increased by 38% with 1.0 mM allantoin under severe drought (Figure 5B). Notable increases in the GR, GPX, and CAT activities by 47%, 58%, and 57%, respectively, were observed with 1.0 mM allantoin under mild drought compared to the drought-only state (Figure 5C–E).

The effects of drought, allantoin, and the interaction of these factors were significant (*p* < 0.01) with regard to Gly I and Gly II activities. The Gly I (42% and 66%) and Gly II (45% and 62%) activities were also reduced when subjected to mild and severe drought compared to the control. However, the application of allantoin at 0.5 mM and 1.0 mM concentrations to the plants further enhanced Gly I and Gly II activities under mild drought compared to the effect of drought only. Meanwhile, under severe drought, the Gly I activity was only increased by 34% with 1.0 mM allantoin application (Figure 5F,G).

### 3.5. Allantoin Improves Plant Growth and Biomass Accumulation under Drought

**Figure 6:**
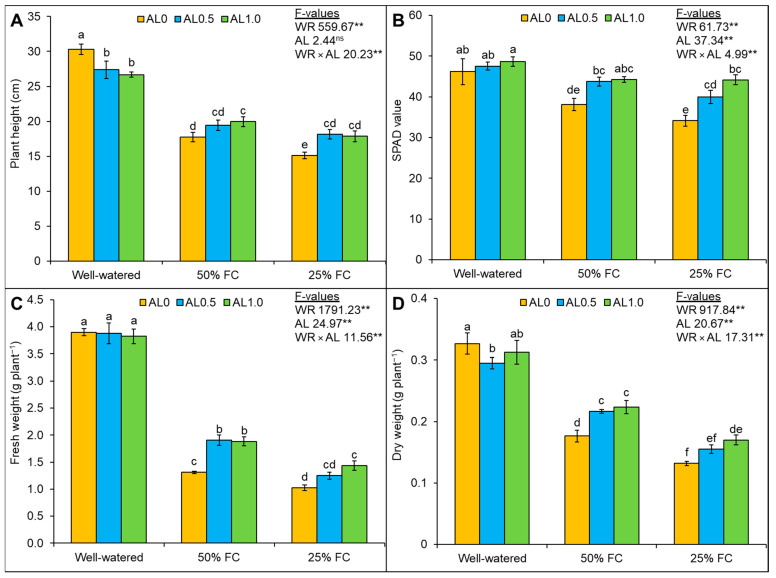
Changes in (**A**) plant height, (**B**) SPAD value, (**C**) fresh weight, and (**D**) dry weight of rapeseed under drought. Here, AL0, AL0.5, and AL1.0 indicate 0, 0.5, and 1.0 mM allantoin, respectively. Mean (±SD) value calculated from three replications and different letters on each bar indicated significant differences among the treatments at *p* ≤ 0.05 after applying Tukey’s HSD test. **: *p* < 0.01, ns: not significant, WR: water regime, AL: allantoin.

Plant height was reduced significantly (*p* < 0.01) by drought. Though the allantoin application was found to be non-significant, the interaction of drought and allantoin was significant (*p* < 0.01). Mild and severe drought caused declines in plant height by 41% and 50%, respectively, compared to the control. Under severe drought, the foliar application of 0.5 mM and 1.0 mM allantoin improved plant height by 20% and 18%, respectively, in comparison to the corresponding effects of drought only, whereas a 13% increase in plant height was observed with 1.0 mM allantoin under mild drought only (Figure 6A). Changes in SPAD values were significant (*p* < 0.01) and caused by either drought or allantoin application, and their interaction was also found to be significant (*p* < 0.01). The SPAD value was reduced upon exposure to mild (17%) and severe (26%) drought compared to control. However, the supplementation of allantoin further enhanced the SPAD value of the plants under mild and severe drought (Figure 6B). The effects of drought, allantoin, and also their interaction were significant (*p* < 0.01) on the FW and DW of the plants. Biomass production was adversely affected upon exposure to drought. The FW declined by 66% and 74% under mild and severe drought, respectively, compared to the control. The supplementation of 0.5 mM and 1.0 mM allantoin increased the FW by 46% and 44%, respectively, under mild drought compared to the effect of drought only, whereas under severe drought, 1.0 mM allantoin only enhanced the FW by 40% (Figure 6C). The DW values of the plants also declined by 46% and 60% under mild and severe drought, respectively, compared to the control, and the supplementation of allantoin further recovered the DW of the drought-affected plants (Figure 6D).

**Figure 7:**
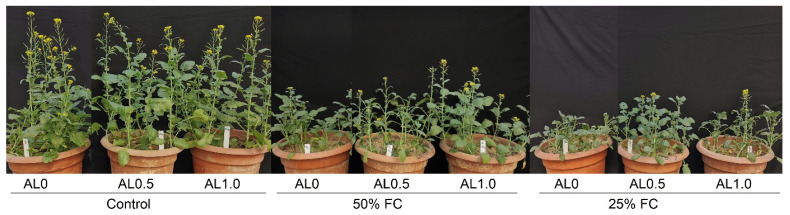
Phenotypic appearance of rapeseed plants under different treatment combinations. Here, AL0, AL0.5, and AL1.0 indicate doses of allantoin at 0, 0.5, and 1.0 mM concentrations that were applied as a foliar spray, and FC denotes field capacity.

The phenotypic appearance of rapeseed plants has been depicted in Figure 7. From the figure, it is noticeable that the growth of plants that were exposed to drought only (without allantoin spray) was hampered. However, the foliar application of allantoin mitigated the osmotic shock caused by drought and improved plant growth compared to the drought-only state (Figure 7).

### 3.6. Effect of Allantoin on the Partitioning Fresh and Dry Weight of Rapeseed under Drought

**Figure 8:**
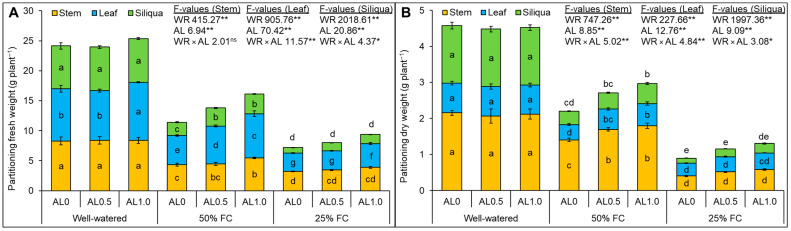
Changes in partitioning, (**A**) fresh weight and (**B**) dry weight of rapeseed under drought. Here, AL0, AL0.5, and AL1.0 indicate 0, 0.5, and 1.0 mM allantoin, respectively. Mean (±SD) value calculated from three replications and different letters on each bar indicate significant differences among the treatments at *p* ≤ 0.05 after applying Tukey’s HSD test. **: *p* < 0.01, *: *p* < 0.05, ns: not significant, WR: water regime, AL: allantoin.

The effects of drought and allantoin application were significant (*p* < 0.01), but their interaction was non-significant on stem FW. Leaf FW was significantly (*p* < 0.01) affected by either drought or allantoin application. The changes in response to drought and allantoin application were significant (*p* < 0.01), however their interaction was also significant at *p* < 0.05. Mild and severe drought adversely affects the FW of the stem, leaf, and siliqua of the plants. Reduction in stem FW by 47 and 61%, leaf FW by 44 and 65%, and siliqua FW by 70% and 87% were found under mild and severe drought, respectively, compared to the control. The 1.0 mM allantoin supplementation increased the stem FW by 26% under mild drought compared to the corresponding drought-only state. Under mild drought, the leaf FW increased by 29% and 51% with 0.5 mM and 1.0 mM allantoin, respectively, compared to the corresponding drought-only state, whereas the siliqua FW increased by 39% and 50%, respectively. The leaf FW was only increased by 1.0 mM allantoin under severe drought by 31% in comparison to the respective drought-only state (Figure 8A).

The changes in DW of stem, leaf, and siliqua were significant (*p* < 0.01) in relation to drought, allantoin application, and their combined effects. The DW of stem, leaf, and siliqua were reduced by 35%, 48%, and 77%, respectively, under mild drought and by 81, 57, and 91%, respectively, under severe drought compared to control. Under mild drought, the application of 0.5 mM allantoin increased the DW of stem, leaf, and siliqua by 21%, 34%, and 20%, respectively, compared to the drought-only state, whereas 1.0 mM allantoin increased them by 28%, 44%, and 50%, respectively. The application of allantoin at either dose did not show a significant increase in DW of stem, leaf, and siliqua under drought (Figure 8B).

### 3.7. Effect of Allantoin on the Yield and Yield Attributes of Rapeseed under Drought

Yield and yield-contributing parameters were influenced when exposed to drought. The changes in number of silique plant^−1^ were significant (*p* < 0.01) and the effects of their combinations were also significant (*p* < 0.05). The numbers of silique plant^−1^ were reduced by 41 and 58% in mild and severe drought, respectively, compared to the control. Foliar supplementation of 0.5 mM and 1.0 mM allantoin increased the silique numbers by 23 and 29% for the mild-drought-affected plants, respectively, whereas a 33% increase in silique number was found with 1.0 mM allantoin application under severe drought (Table 2). Silique length was significantly (*p* < 0.01) affected by drought, allantoin application, and their combined effects. The silique length declined by 33% and 54% for the mild- and severe-drought-affected plants, respectively, compared to the control. The 1.0 mM allantoin application to the mild- and severe-drought-affected plants showed improvement in the silique length by 29% and 33%, respectively, compared to the control. On the other hand, 0.5 mM allantoin only increased the length by 23% under mild drought (Table 2). The number of seeds silique^−1^ was affected significantly (*p* < 0.01) by drought and allantoin application and their combined effects was also significant (*p* < 0.01). A similar reduction was also observed in the case of the number of seeds silique^−1^ under mild (45%) and severe (72%) drought compared to the control. However, the supplementation of 1.0 mM allantoin increased the number of seeds silique^−1^ under mild and severe drought by 21% and 44%, respectively, in comparison to the corresponding drought-only state (Table 2). The effects of drought, allantoin application, and their combined effects were significant (*p* < 0.01) on the 1000-seed weight and seed yield plant^−1^ of rapeseed. One-thousand-seed weight declined by 34% and 49% under mild and severe drought, respectively, compared to the control. Reduction in the seed weight ultimately affects the seed yield of the drought-affected plants, which was decreased by 50% and 71% under mild and severe drought, respectively. However, the supplementation of 0.5 mM and 1.0 mM allantoin increased the thousand-seed weight as well as the seed yield plant^−1^ under mild drought, whereas the 1.0 mM allantoin performed better in the case of increasing these parameters under severe drought (Table 2).

### 3.8. Correlation among Oxidative Stress Indicators, Antioxidant Defense System, Growth, and Yield Attributes of Rapeseed

**Figure 9:**
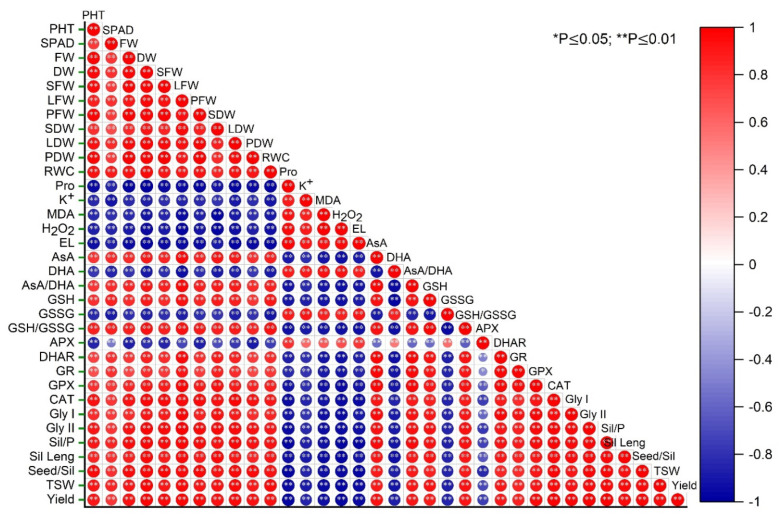
Matrix represents the correlation among the different observed attributes of rapeseed under drought. Here, SPAD: soil and plant analysis development, PHT: plant height, FW: fresh weight, DW: dry weight, SFW: stem fresh weight, LFW: leaf fresh weight, PFW: pod fresh weight, SDW: stem dry weight, LDW: leaf dry weight, PDW: pod dry weight, Pro: proline, K^+^: potassium ion, MDA: malondialdehyde, EL: electrolyte leakage, H_2_O_2_: hydrogen peroxide, AsA: ascorbate, GSH: glutathione, DHA: dehydroascorbate, GSSG: glutathione disulfide, APX: ascorbate peroxidase, GR: glutathione reductase, DHAR: dehydroascorbate reductase, CAT: catalase, GPX: glutathione peroxidase, Gly I: glyoxalase I, Gly II: glyoxalase II, and TSW: thousand-seed weight.

The heat map (Figure 9) represents the correlation analysis of different observed parameters under drought. The matrix shows that oxidative stress markers (MDA, H_2_O_2,_ and EL) are negatively linked with AsA and GSH, whereas they are positively related to DHA and GSSG. Antioxidant enzyme (except APX) activities have a significant negative relationship with the stress markers. A similar negative link was also found with the components of glyoxalase enzymes (Gly I and Gly II). In addition, the RWC was negatively linked, and the proline and K^+^ contents were positively linked with stress-indicating components. Furthermore, the proline, K^+^, and oxidative stress markers are positively correlated with the growth and yield attributes.

## 4. Discussion

Proline is an amino acid intimately linked with osmotic adjustment, photosynthesis, and nutrient availability and has a signaling role in modulating gene expression and metabolic processes under stress [55]. Plants accumulate such osmolytes (proline) to protect their cellular organelles from the disruptive effect of water stress [56]. Drought led to an increase in the accumulation of proline as an osmoprotectant to eradicate osmotic shock (indicated by lower RWC) in our experiment. Reduced RWC in chickpea accelerated proline contents under drought, reported by Ahmed et al. [57]. Increased levels of proline were previously documented in other crops too, i.e., rice [58] and maize [59], etc., which aligns with the findings of the present study. Wu et al. [60] demonstrated that the contents of proline, allantoin, and sucrose were increased in *Suaeda salsa*, adjusting osmoregulation. *Selaginella lepidophylla* accumulated an abundant amount of allantoin in a state of dehydration, indicating a protective role in survival and recovery from stress [61]. Tolerant cultivars of sesame showed profound levels of proline, arginine, lysine, and allantoin in plants under drought compared to the susceptible cultivars [62]. Recently, Wang et al. [63] reported that exogenous allantoin reduced proline in plants under salinity. Thus, these findings indicate a direct link between allantoin and osmoregulation. Moreover, allantoin has an intrinsic role in accumulating a stress-responsive hormone, abscisic acid (ABA), under water stress [18]. Allantoin application upregulated the ABA biosynthesis gene, *OsUGT85E1,* which accelerates the accumulation of ABA, induces stomatal closure, and enhances the ROS scavenging capacity of rice under drought [64]. Allantoin also plays a key role in synthesizing jasmonic acid under drought through the ABA pathway and improved resistance to drought, as reported by Lu et al. [65]. Potassium ions regulate stomatal opening and closing to reduce water losses through stomata under drought [66], and their accumulation was lowered upon allantoin treatment in our experiment. Thus, exogenous allantoin in the current study mitigated proline and K^+^ contents and enhanced the RWC of the plants, corroborating the findings that it has a role in maintaining osmotic adjustment and improving resistance to stresses.

Drought is responsible for hampering photosynthesis and accelerating photorespiration in plants. Thus, an excessive amount of ROS accumulates at the cellular level, and the ruthless effect of ROS disrupts biomolecules, viz., proteins, lipids, and nucleic acids [67]. Disruption of the cellular organelles and membrane fluidity results in the distortion of structural integrity and increased electrolyte leakage, resulting in fast desiccation and cell death [68]. In our experiment, the H_2_O_2_ content as well as lipid peroxidation (MDA levels) and EL of the rapeseed plants were notably increased when exposed to drought. Drought also showed an upregulated ROS generation in rice, previously recorded by Lu et al. [65]. However, exogenous allantoin mitigated H_2_O_2_, MDA, and EL of the drought-stressed plants in our experiment. Allantoin performs an intermediate role in ureide metabolism to protect plants from oxidative stress. Ureides are important nitrogen-rich metabolites that are derived from the oxidative degradation of purines. The conversion of xanthine to uric acid is mediated by xanthine hydrogenase, after which the products are transported to peroxisomes, where they are oxidized to hydroxyisourate and subsequently to allantoin by urate oxidase and allantoin synthase, respectively [69,70]. Allantoinase further hydrolyzes allantoin to allantoic acid in the endoplasmic reticulum, where subsequent catabolic reaction decomposes purine rings to produce glyoxylate, carbon dioxide, and four units of ammonium ions [69]. Ureide breakdown is not only attributed to recycling nitrogen from purines but is also considered a potential protectant against ROS [71,72]. Previous studies also demonstrated that the accumulation of allantoin and allantoate accelerates the ureide metabolic pathway and mitigates the ability of ROS to confer various stresses [26,63,73]. Lu et al. [65] demonstrated that drought escalated ROS production in rice, but the ROS were decreased when the seeds were grown in allantoin-incorporated growing media. The RNAi *xanthine dehydrogenase* mutants were reported to be more susceptible to drought [74]. Suppression of the *xanthine dehydrogenase* gene led to the increased accumulation of H_2_O_2_ in Arabidopsis, thus consequently increasing cell death in the mutants. But surprisingly, when the growth media was exogenously supplied with ureic acid, drought tolerance and improved chlorophyll contents and biomass weight were demonstrated [74]. Irani and Todd [28] showed Arabidopsis tolerance to salinity due to the positive effect of allantoin on reducing O_2_^•–^ and H_2_O_2_ in the mutants. The limited supply of water also induces higher allantoate accumulation in *Phaseolus vulgaris* and enhances the plant’s tolerance to stress [75]. Under salt stress, the application of allantoin reduced the H_2_O_2_, O_2_^•−^, and MDA contents in sugar beet plants, also reported by Liu et al. [40]. These findings indicate a positive impact of allantoin in reducing oxidative damage in different plants, which corroborates our results.

Drought induces stomatal closure, decreased CO_2_ assimilation, and hampered photosynthetic rate and phytochemistry of chloroplasts, causing the overaccumulation of ROS. Though ROS are known for transporting signals to the nucleus from various organelles through the mitogen-activated protein kinase pathway (MAPK), acting as signaling molecules or secondary messengers to enhance tolerance against stresses, excess ROS production causes cellular damage and leads to oxidative bursts in plants. Thus, plants rely on their intrinsic mechanism of antioxidant defense systems composed of enzymatic and non-enzymatic antioxidants to scavenge ROS and reduce ROS-induced oxidative damage [76]. The non-enzymatic components of antioxidant defense, AsA, and GSH are engaged in counteracting uncontrolled ROS-producing cascades along with other antioxidant enzymes in a coordinated manner. To scavenge H_2_O_2_, AsA is one of the most potent substrates that donates electrons to APX. After adopting an electron, APX converts H_2_O_2_ into water, and the AsA is also transformed into monodehydroascorbate (MDHA). The activity of MDHAR again regenerates AsA from MDHA, and a portion of the enzyme converts to DHA [77]. Drought caused a notable reduction in the AsA and AsA/DHA ratio in plants in the present study, which also supports the previous results [57,78]. Allantoin has been considered an indicator of oxidative stress markers and has a dual role in antioxidant activities and nitrogen recycling [73]. Moreover, it functionally resembles vitamins [79]. Exogeneous allantoin increased the AsA and reduced DHA, therefore upregulating the AsA/DHA ratio of the plants under drought. Previously, Dresler et al. [24] also reported that allantoin application at higher doses (100 and 1000 μM) increased the AsA content in cadmium-stressed *Cucumis sativus* plants. Similarly, Liu et al. [40] also found that allantoin application to salt-stressed plants increased AsA levels notably. Later on, the GSH helps DHA produce AsA again, and oxidation of GSH further results in the production of GSSG. The activity of GR and the role of NADPH as an electron donor regenerates GSH from GSSG in a cyclic manner [80]. The GSH content in plants decreases when the plants are exposed to dehydrated conditions, but the allantoin application further enhances GSH and reduces GSSG levels, thus helping uplift the GSH/GSSG ratio in plants. Allantoin was also found to increase GSH contents in the leaves of sugar beet seedlings under salt stress [40]. Besides the AsA-GSH cycle, other antioxidant enzymes are involved with GSH, for example GPX, which is a highly reactive thiol group that uses GSH and other thioredoxins to scavenge organic hydroperoxides and H_2_O_2_ [81]. Catalase is a tetrameric heme-containing enzyme that transforms H_2_O_2_ to H_2_O, and in the absence of this enzyme in chloroplasts, APX is highly active in diminishing H_2_O_2_ production [82]. CAT and APX were found to increase with allantoin application upon exposure to salt stress, reported by Liu et al. [40]. Subsequently, the toxic effect of H_2_O_2_ was eliminated by converting it into H_2_O; as a result, APX and CAT activities increased in the allantoin-treated plants. GPX also helps detoxify H_2_O_2_ and xenobiotic compounds; thus, the activities of GPX increased with allantoin. The allantoin-induced enhancement of GPX activities activated GSH-dependent peroxide detoxification systems in order to provide enhanced tolerance against drought- mediated harmful byproducts [83]. Allantoin was also shown to enhance APX and GPX activities under cadmium stress [24]. Dawood et al. [25] also found that the activities of APX, CAT, and GPX were accelerated with the application of allantoin under ultraviolet-C stress. Thus, the findings of previous studies are also supported by our present study: allantoin increases the non-enzymatic (AsA and GSH) and enzymatic components of the antioxidant defense system to mitigate ROS damage.

Under stress, plants accumulate another harmful cytotoxic compound, MG, that is produced as a derivative of different metabolic pathways. The toxic effect of MG distorts the membrane structure and injures cellular organelles [13]. Thus, the harmful effect of MG is mitigated with the activity of Gly I and Gly II in a two-step process where GSH plays a key role. Here in the present experiment, the MG content of plants was increased upon exposure to drought due to the reduced activity of Gly I and Gly II enzymes. This activity of Gly I and Gly II was also found in previous studies [57]. In MG detoxification, firstly, conversion of MG to SLG occurs with the help of Gly I, which uses GSH as a cofactor of the glyoxalase system. In the last step, SLG is converted to D-lactic acid and regenerates GSH with the help of Gly II enzymes [14]. Allantoin application to drought-affected plants showed a higher level of GSH accumulation, thus upregulating the Gly I and Gly II activities in rapeseed plants. The increased activities of Gly I and Gly II in plants ultimately mitigate MG-induced damage in plants.

**Figure 10:**
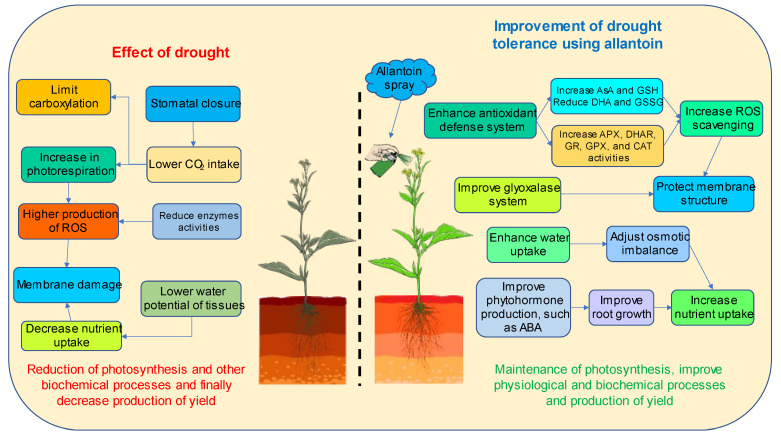
Possible mechanism of allantoin in mitigating drought-induced damages in plants.

Drought leads to an alteration in the plant–water relationship, causing dehydration due to an increase in water losses from the tissues in plants. This water loss ultimately promotes osmotic stress, suppresses mitotic cell division, inhibits cell enlargement, and reduces photosynthesis in plants [84,85]. Besides this, drought adversely affects stomatal conductance, carbon dioxide diffusion, electron transport, water use efficiency, respiration, transpiration, and membrane function in plants [86]. All of these are involved in the impairment of plant growth and biomass production (both FW and DW). Our findings reveal a notable reduction in plant height, SPAD, FW, and DW upon exposure to drought, which is aligned with the previous reports of Ahmed et al. [57], Wasaya et al. [87], and Mohi-Ud-Din et al. [88]. Under water deficit conditions, nitrogen fixation is restricted due to lower photosynthate supply or reduction in oxygen flux, or lack of deposition of nitrogenous compounds in plants. Hence, the inhibition of nitrogen fixation promotes ureide (allantoin and its hydrolyzed product allantoate) metabolism in various tissues through an allantoate-degrading enzyme, allantoate amidohydrolase (AAH) [74]. AAH converts allantoin to allantoate, and through the hydrolysis of the internal amide bond, allantoate is converted to urea and ureidoglycolate. The further conversion of ureidoglycolate yields glyoxylate and urea. The activity of urease enzymes hydrolyzes urea to produce ammonia and carbon dioxide and supports the growth and development of plants [69,75]. In the present study, exogenous allantoin improved the growth and biomass accumulation of rapeseed plants under drought. Dresler et al. [24] demonstrated that exogenous allantoin improved shoot biomass and photosynthetic (chlorophyll *a*) accumulation in plants. Allantoin releases more ammonium ions to plants upon complete breakdown [18], and nitrogen (as a micronutrient) is a vital component for proteins, lipids, nucleic acids, certain hormones (i.e., indole-3-acetic acid, cytokinin), and chlorophylls [89]. Moreover, several studies also supported that nitrogen or nitrogenous compounds could mitigate the detrimental effect of stress in different crops [90,91]. In a nutshell, the application of allantoin improved AsA and GSH contents with a concomitant reduction in DHA and GSSG in plants and rejuvenated the ratios of AsA/DHA and GSH/GSSG under drought conditions. Besides this, the activities of antioxidant enzymes such as APX, DHAR, GR, GPX, and CAT, as well as glyoxalase enzymes (Gly I and Gly II), were also increased from allantoin application. Thus, a consequent reduction in H_2_O_2_ production and lipid peroxidation was also observed in rapeseed plants. Moreover, allantoin also increased the RWC and reduced proline content in plants under drought, thus ultimately improving the plant growth and yield attributes of rapeseed (Figure 10).

## 5. Conclusions

The findings of the present study highlight the protective role of allantoin in mitigating drought-induced oxidative stress in rapeseed, leading to improvement in plant growth, physiology, and biochemical attributes. Based on the obtained results, it is noticeable that the application of allantoin increased the performance of plants under drought by improving the activity of non-enzymatic (AsA and GSH) and enzymatic (APX, DHAR, GR, CAT, and GPX) antioxidants. Allantoin also improved glyoxalase enzyme activity (Gly I and Gly II) in plants. Thus, improvement in antioxidant defense and the glyoxalase system helps in ROS scavenging and MG-induced damages and increases RWC and chlorophyll content, which ultimately enhance plant growth, biomass production, and yield of rapeseed under drought. Our findings demonstrate a cumulative effect of allantoin in enhancing antioxidant defense and stress tolerance in plants. While the primary focus of this study was to investigate the response of rapeseed to allantoin application in terms of antioxidants, we acknowledge that a genomic and phytohormonal (ABA, jasmonic acid) analysis, as conducted in other studies [28,92], could offer additional insights into the molecular mechanisms of allantoin’s action in rapeseed.

Therefore, further investigations, specifically examining the expression of stress-responsive genes and phytohormone activity, are warranted to gain a deeper understanding of how allantoin is associated with the antioxidant defense system and signaling pathways related to drought tolerance. Additionally, it would be valuable to explore the functional role of allantoin in plants under stress conditions by utilizing allantoin inhibitors in future studies. Such research endeavors can provide valuable knowledge and contribute to a comprehensive understanding of allantoin’s potential benefits in enhancing plant resilience to environmental stress.

## Figures and Tables

**Table 2:** Changes in the yield and yield-contributing attributes, viz., no. of silique plant^−1^, silique length, no. of seeds silique^−1^, 1000-seed weight, and seed yield of rapeseed under drought. Here, AL0, AL0.5, and AL1.0 indicate 0, 0.5, and 1.0 mM allantoin, respectively. Mean (±SD) value calculated from three replications and different letters indicate significant differences among the treatments at *p* ≤ 0.05 after applying Tukey’s HSD test. **: *p* < 0.01, *: *p* < 0.05, WR: water regime, AL: allantoin.

Water Regimes (WRs)	Allantoin (AL) Doses	No. of Silique Plant^−1^	Silique Length (cm)	No. of Seeds Silique^−1^	1000-Seed Weight (g)	Seed Yield (g Plant^−1^)
Well-watered	0 mM	39.33 ^a^ ± 1.72	4.60 ^a^ ± 0.28	22.53 ^a^ ± 0.93	2.60 ^a^ ± 0.10	2.28 ^a^ ± 0.11
	0.5 mM	40.07 ^a^ ± 2.04	4.25 ^ab^ ± 0.40	22.40 ^a^ ± 0.82	2.47 ^a^ ± 0.06	2.26 ^a^ ± 0.02
	1.0 mM	42.67 ^a^ ± 0.12	4.30 ^ab^ ± 0.21	22.16 ^a^ ± 1.88	2.47 ^a^ ± 0.09	2.17 ^a^ ± 0.11
50% FC	0 mM	23.17 ^d^ ± 0.68	3.07 ^c^ ± 0.10	12.37 ^c^ ± 0.50	1.72 ^c^ ± 0.11	1.14 ^d^ ± 0.04
	0.5 mM	27.87 ^c^ ± 1.22	3.78 ^b^ ± 0.21	14.20 ^bc^ ± 0.60	2.13 ^b^ ± 0.13	1.34 ^c^ ± 0.04
	1.0 mM	32.98 ^b^ ± 2.39	3.95 ^b^ ± 0.09	14.97 ^b^ ± 0.55	2.18 ^b^ ± 0.04	1.80 ^b^ ± 0.12
25% FC	0 mM	16.41 ^f^ ± 0.59	2.23 ^e^ ± 0.07	6.40 ^e^ ± 0.20	1.33 ^d^ ± 0.06	0.66 ^f^ ± 0.01
	0.5 mM	18.90 ^ef^ ± 0.26	2.41 ^de^ ± 0.11	8.30 ^de^ ± 0.20	1.57 ^cd^ ± 0.08	0.76 ^ef^ ± 0.05
	1.0 mM	22.34 ^de^ ± 1.16	2.96 ^cd^ ± 0.18	9.22 ^d^ ± 0.34	1.71 ^c^ ± 0.06	0.91 ^e^ ± 0.02
F-values	WR	568.64 **	176.52 **	687.55 **	292.41 **	980.07 **
	AL	49.78 **	9.74 **	9.83 **	18.37 **	33.52 **
	WR × AL	4.39 *	8.65 **	3.59 *	12.89 **	24.04 **

## Data Availability

All data are available in this article.
